# Supplementary material for: Comparative multiomics analysis of cell physiological state after culture in a basket bioreactor
Source: Sci Rep. 2022 Nov 23;12:20161. doi: 10.1038/s41598-022-24687-4 (PMC9686226; doi:10.1038/s41598-022-24687-4)

47702354:-@NW_023666041.1:147701506:147701556:-@NW_023666041.1:147695028:147695090:-@NW_02366

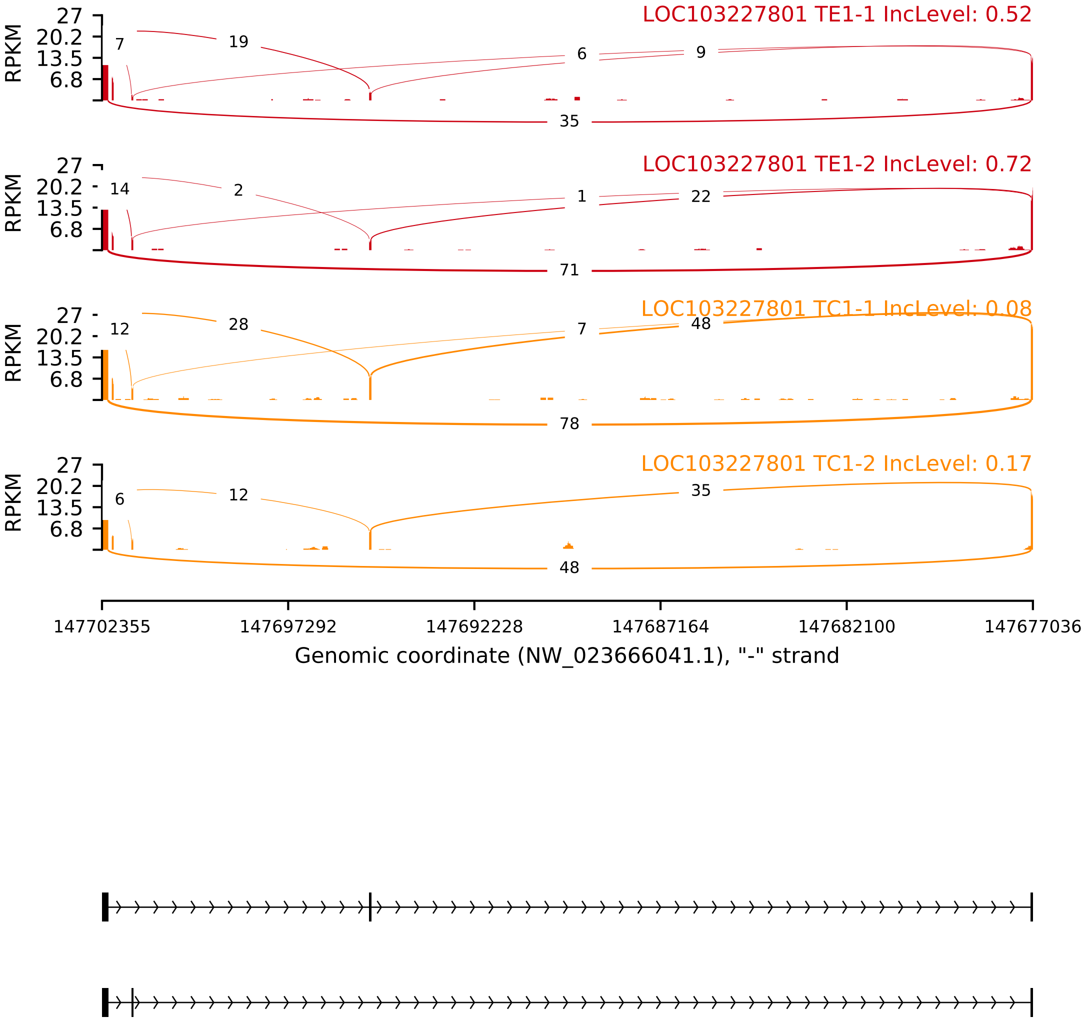

Supplement: Supplementary file 1 — Supplementary Information 1. [file 41598_2022_24687_MOESM1_ESM.zip › raw data/Transcriptomic raw data/7.AS/2.dasplot/TE1vsTC1/MXE/10_LOC103227801_NW_023666041.1_147702186_147702354_-@NW_023666041.1_147701506_147701556_-@NW_023666041.1_147695028_147695090_-@NW_023666041.1_147677034_147677093_-.pdf]
